# IGBT Overcurrent Capabilities in Resonant Circuits

**DOI:** 10.3390/s24237631

**Published:** 2024-11-29

**Authors:** Basil Mohammed Al-Hadithi, Miguel Jimenez

**Affiliations:** 1 Intelligent Control Group, Centre for Automation and Robotics, Universidad Politécnica de Madrid, UPM-CSIC, C/J. Gutierrez Abascal, 28006 Madrid, Spain; basil.alhadithi@upm.es; 2 Department of Electrical, Electronics, Control Engineering and Applied Physics, Higher Technical School of Industrial Design and Engineering, Universidad Politécnica de Madrid, C/Ronda de Valencia, 28012 Madrid, Spain; 3 Higher Technical School of Industrial Design and Engineering, Universidad Politécnica de Madrid, C/Ronda de Valencia, 28012 Madrid, Spain

**Keywords:** MOSFET, IGBT, zero current switching, IGBT driver, resonant circuits, overcurrent sensor

## Abstract

The control of IGBT (insulated gate bipolar transistor) and MOSFET (metal oxide semiconductor field effect transistor) is of great interest nowadays as they are widely used in electric vehicles, photovoltaic applications, and a multitude of systems. The field of power electronics and their correct activation ensures that the transistors are operated without being destroyed. In this work, a double resonant transformer was built and used to produce very high currents. These currents are switched by a full bridge of resonant IGBT transistors to demonstrate the feasibility of exceeding the maximum permissible transistor currents in a resonant system. The system is controlled by the feedback from two current sensors. In this case the currents exceed in a 170% the peak current of the transistor without problems. In this way, resonant circuits with IGBT transistors can be designed with currents lower than the maximum currents of the resonant circuit, therefore reducing the cost of the circuit and reducing the switching losses to nearly zero.

## 1. Introduction

The use of power electronics is widespread around the world, and its application continues to expand into new areas daily. Currently, the use of power transistors like IGBTs and MOSFETs allows for the rapid switching of any device with very low losses.

On the one hand, MOSFET transistors allow for very fast switching, as seen in [1,2], where frequencies of hundreds of kHz and even MHz can be achieved with relatively low switching losses by employing technologies like SiC-MOSFETs. These types of transistors can reach high voltages, up to the kV range [3], but the allowable currents are significantly lower, around 50 A. On the other hand, IGBT transistors can handle much higher currents with voltages in the same range, as seen in [4], where a 1 kA and 2.5 kV IGBT module is developed. However, the main problem with these transistors is their switching frequencies, as manufacturers do not recommend exceeding 20 kHz. This is due to the high switching losses at higher frequencies, which could potentially destroy the transistors [5,6,7].

**Figure 1:**
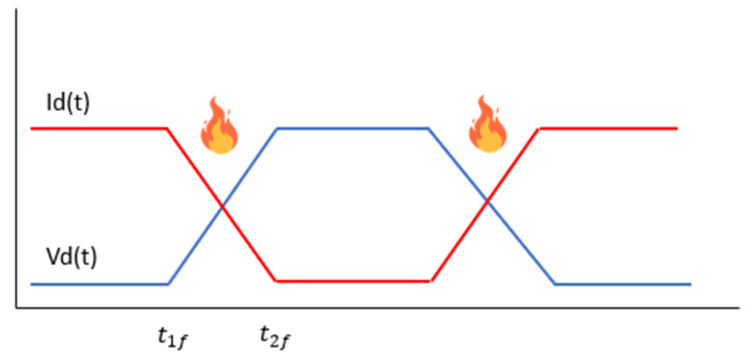
IGBT transistor conduction and switching losses without ZCS control.

These switching losses occur because, during the opening or closing of the transistor, the voltage and current do not simultaneously drop to zero, leading to losses, as shown in Figure 1. Between times $t_{1f}$ and $t_{2f}$, the collector current and the collector–emitter voltage cause switching losses, which are directly dependent on frequency.

IGBT transistors are used in a wide range of high-power devices. Their use is notable in converters and inverters, as seen in [8,9], where powers of several kW are developed, although the frequencies are relatively low compared to MOSFET technologies. As a result, new switching techniques, such as Soft-Switching techniques, have been developed to allow the use of Zero Current Switching (ZCS) or Zero Voltage Switching (ZVS) to switch transistors exactly at the zero crossing of current or voltage, thus reducing switching losses and increasing frequency. In [10], this ZCS technology is used to achieve frequencies higher than 60 kHz without damaging the transistors.

**Figure 2:**
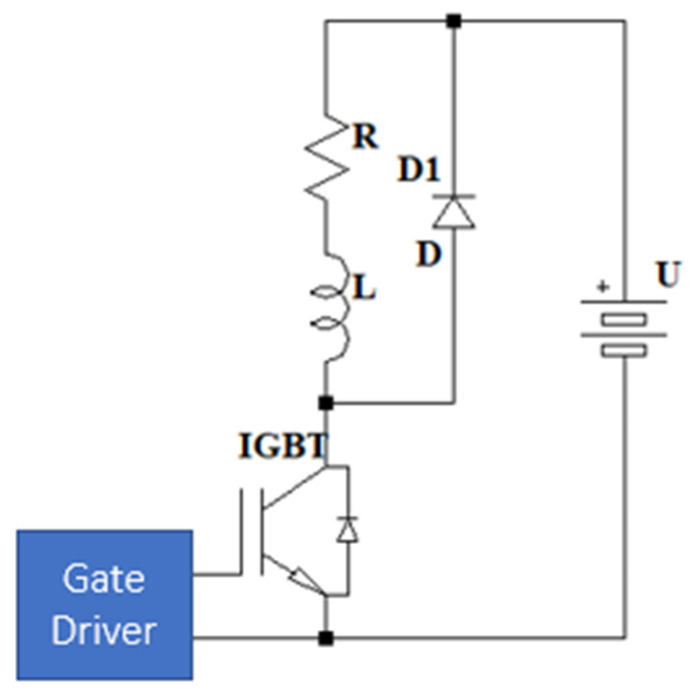
IGBT gate driver in a transistor with an inductive load and freewheel diode.

These IGBT transistors require a driver (Figure 2) capable of supplying sufficient current to the gate to bias the transistor and enable conduction. This gate current can be relatively high when the current passing through the transistor is significant and has a relatively high RMS value when the switching frequency exceeds 20 kHz. In [11,12,13], several of these drivers are developed, capable of providing the necessary current to the transistor and switching at high frequencies using soft-switching techniques.

Converters and inverters require high reliability in the components. Manufacturers establish certain safety conditions and maximum operating parameters, such as voltage, current, and frequency, which have been extensively studied, as in [14,15]. However, in certain situations, such as resonance, transistors are subject to higher demands, significantly increasing the cost.

In [16,17], it is shown that in resonant circuits, the allowable current of the transistor depends on its temperature, although for safety reasons, the maximum currents specified by manufacturers are not exceeded. On the other hand, in some studies, such as [18,19], this current has been momentarily improved by using high frequencies, but the maximum currents specified by manufacturers are never exceeded to avoid damaging the transistors. Many studies have also been conducted to actively protect these transistors from high currents, as seen in [20,21], where the transistor’s gate voltage is used as a reference in case of overcurrent in the module, stopping the switching process and preventing destruction. Other current investigations focus on short circuits currents that could also destroy the transistors [22,23,24,25]. In the case of [25], the nominal currents of the transistors are exceeded for time periods shorter than 10 µs; however, the current is only exceeded during the transistor’s turn-off and not during its turn-on.

This work presents an effective method to avoid oversizing power transistors under resonant conditions, allowing them to handle turn-on and turn-off transient peak currents and frequencies higher than nominal, thus withstanding stress conditions far beyond those specified by manufacturers. To achieve this, soft-switching techniques are used, specifically ZCS, to switch the transistors using a specially designed driver. To achieve high currents and voltages for testing the IGBT transistors, a high-frequency resonant circuit has been built, based on a solid-state Tesla Coil [26] and its respective control circuits to switch the transistors.

The work begins with a description of the high-power resonant circuit used for testing, as well as the necessary drivers in the Section 3. In the Section 3 the system is simulated to analyze the maximum currents that the prototype can withstand under resonance. In Section 4 results of the current analysis in the transistors are presented.

## 2. System Construction

**Figure 3:**
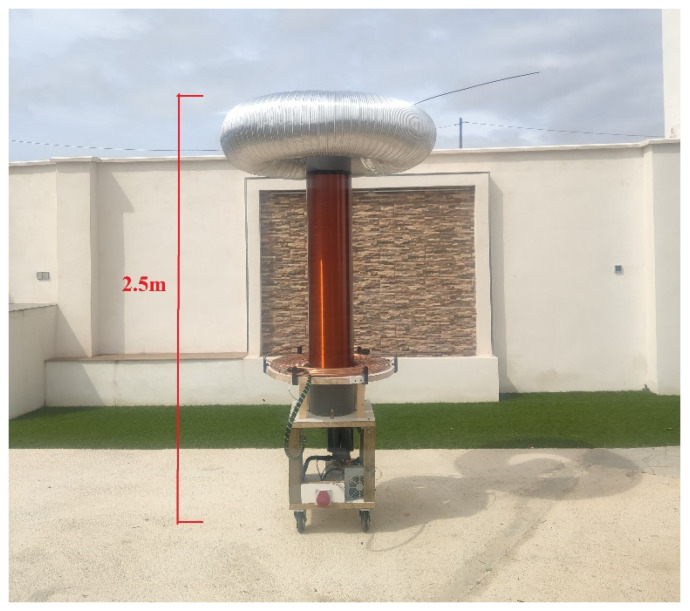
Tesla coil of 2.5 m built for this work with topload.

This work uses a doubly resonant transformer to elevate the grid voltage to very high levels, achieving efficiencies greater than 90% [27]. It is a Double Resonant Solid Stage Tesla Coil (DRSSTC) that employs IGBT-type transistors to drive a resonant circuit. The prototype has a nominal power of 10 kW, allowing for the testing of power transistors at peak currents exceeding 1000 A in resonance. This circuit can be seen in Figure 3.

**Figure 4:**
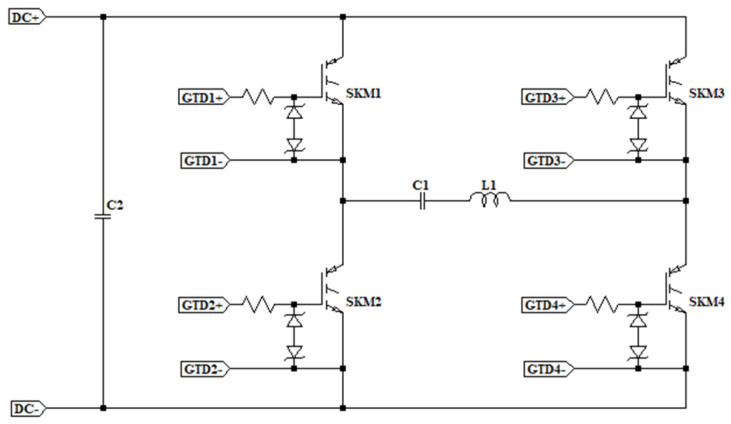
DRSSTC primary diagram based on four IGBT transistors and their gate driver protection circuits.

The operation of a Tesla Coil is based on having two resonant RLC circuits coupled through an air core. The primary of the resonant circuit is connected to an H-bridge of IGBT transistors that are capable of switching through a control circuit. This circuit receives feedback from the current via a current sensor in the primary winding to generate signals and adjust the frequency. In contrast to classical tesla coils, this type of tesla coil employs IGBT transistors to switch the current at the resonant frequency instead of a spark gap, allowing the current and frequency to be adjusted for more efficient operation and higher circuit currents. The operating schematic of the DRSSTC used in this project can be seen in Figure 4, the power stage is mainly composed of the transistor H-bridge and its gate protection circuits, capacitor C1 and coil L1 form the primary resonant circuit, while C2 is the DC filter capacitor, the following sections explain the prototype used in more detail.

The selection of this type of resonant circuit over others is due to its capability to achieve extremely high currents and frequencies, reaching hundreds of amperes in the primary circuit through resonance phenomena. Achieving these reference levels is particularly challenging with other equipment, such as induction heaters [28,29,30], which are typically designed for lower currents and frequencies and tend to have higher associated costs. 

### 2.1. Resonant Circuits

**Figure 5:**
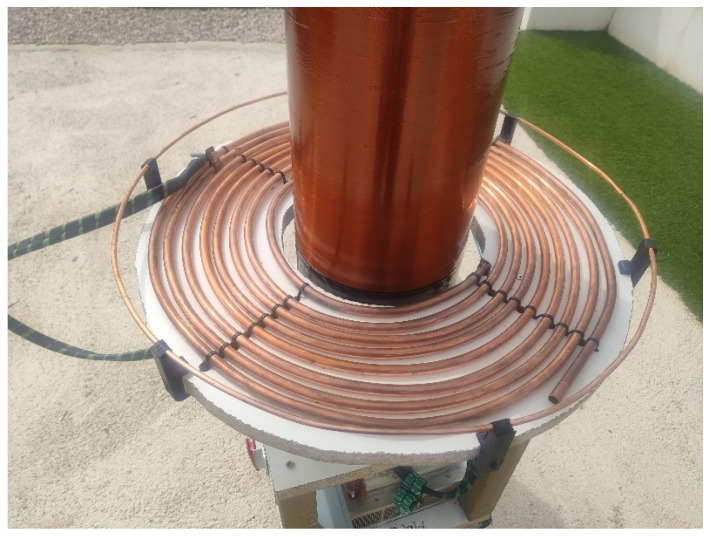
Primary resonant circuit based on a flat concentrical copper tube and secondary resonant circuit with vertical coil.

The resonant circuits are magnetically coupled through an air core [31] at a frequency of 37 kHz with a low coupling coefficient of 0.14. The primary resonant circuit consists of a 0.66 uF capacitor and an 8-turn coil with an inductance of 28 uH. The secondary circuit is formed by a coil of 2100 turns with an inductance of 258 mH [32] and the parasitic capacitance of the turns and a toroid at the top point, totaling 44 pF. Both systems are designed and calculated to operate at a design frequency of 37 kHz. As shown in Figure 5, the circuits are not coplanar, which results in a low coupling coefficient, thereby reducing the energy transfer from the primary to the secondary.

### 2.2. H Bridge

**Figure 6:**
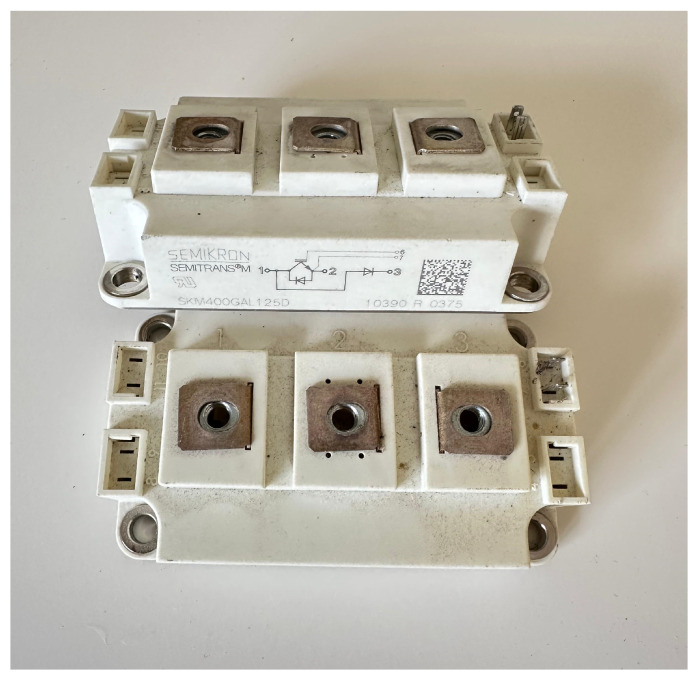
Transistors IGBT SKM400 with high current terminals.

The inverter bridge is the stage that converts direct current into alternating current to supply the resonant circuit. It consists of a full bridge made up of four IGBT transistors, which are used for testing purposes. These transistors are of the SKM type for high-power applications, and their size can be seen in Figure 6.

**Figure 7:**
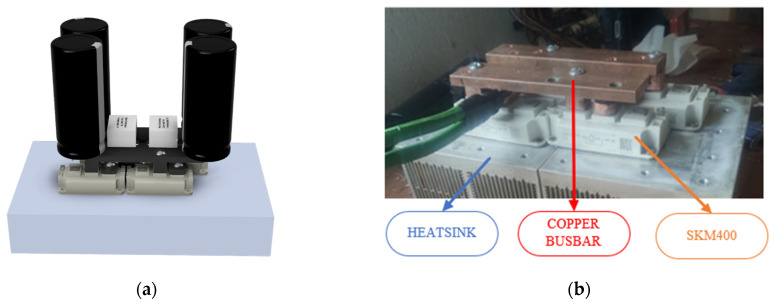
Inverter bridge assembled. (**a**) Three-dimensional design of the inverter bridge module based on a compact configuration with low inductance. (**b**) Assembly of the inverter bridge using copper plates and aluminum heatsinks.

The full bridge has been designed using a 3D process that minimizes material usage and facilitates heat dissipation (Figure 7a). The transistors are mounted using 5 mm thick copper plates to minimize resistance and impedance in the system, allowing for high currents in the circuit, as shown in Figure 7b.

**Figure 8:**
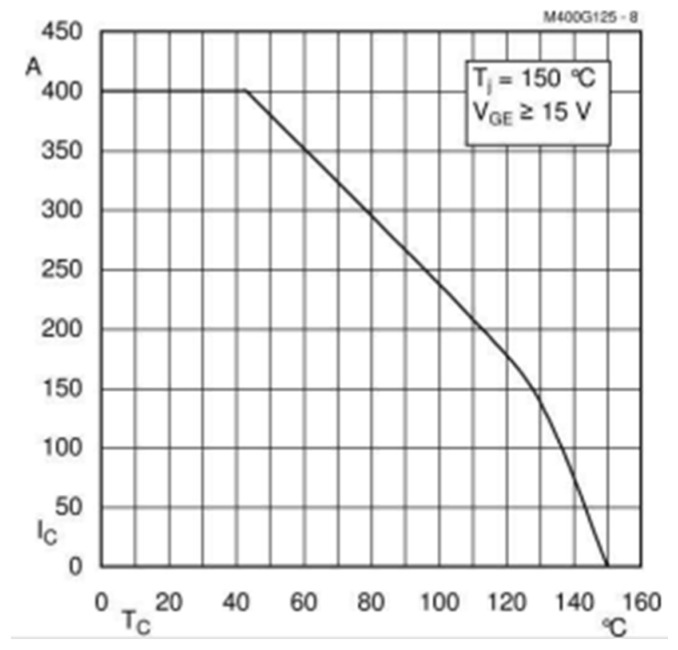
Nominal current of the SKM400 with temperature.

The IGBTs used are of the SKM400GAL125 type, with a nominal current of 400 A and a peak of 600 A at 25 °C. However, this is reduced to a nominal current of 300 A with a transistor temperature of 80 °C, which is typically the operating temperature for this type of device in power electronic circuits. Figure 8 shows the dependence of the transistor current on the device temperature.

### 2.3. Power Supply

To power the system, a rectifier with a voltage doubler is used, converting 230 V AC to achieve a voltage of 511 V DC to supply the transistors. This system includes an output filter consisting of a 6800 uF capacitor capable of providing the resonant current to the transistors.

### 2.4. Feedback and Overcurrent Sensors

These sensors are used to supply the control of the system and generate a phase-aligned signal at the resonance frequency determined by the Tesla Coil. Additionally, they are employed to detect and limit the current in the resonant circuit, preventing it from exceeding damaging levels for the system.

**Figure 9:**
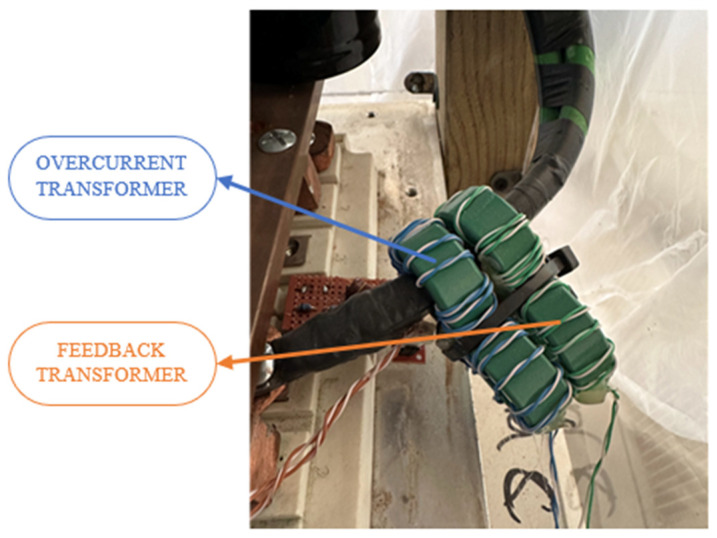
Feedback and overcurrent transformers in the primary circuit wire between IGBT and primary coil.

First, since the currents are on the order of 1000 A, two current transformers are used in cascade, both with a ratio of 32:1, resulting in a total transformation ratio of 1024:1, allowing for the generation of a current that can be easily detected. The transformers are designed with a ferromagnetic core measuring 36 × 23 × 15 mm and a relative magnetic permeability of 8000 to avoid saturation at high frequencies, as shown in Figure 9.

**Figure 10:**
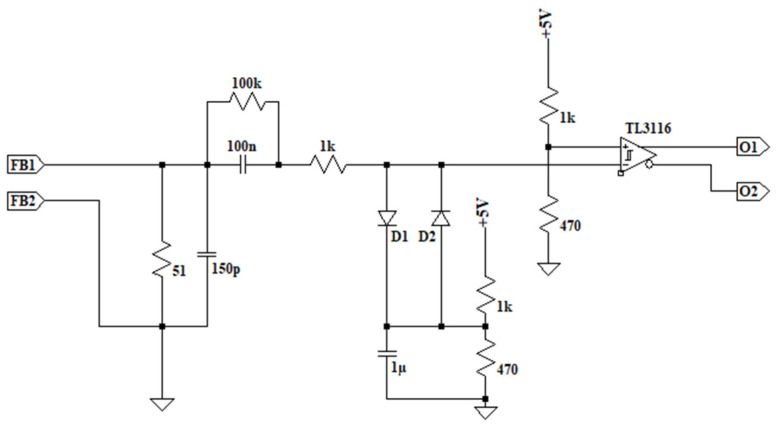
Feedback current sensor for ZCS with reference fast comparator and TTL signal output.

The output from the transformers will be approximately 1 A with a frequency of 37 kHz. To achieve an accurate measurement and generate a square wave signal, the current is passed through a 51 Ω resistor. Using two Schottky diodes and a voltage divider, the signal is conditioned to provide a 5 V reference. This, combined with a fast comparator and another reference signal, generates a TTL signal of 5 V that is in phase with the input current. The simplified design schematic is represented Figure 10.

**Figure 11:**
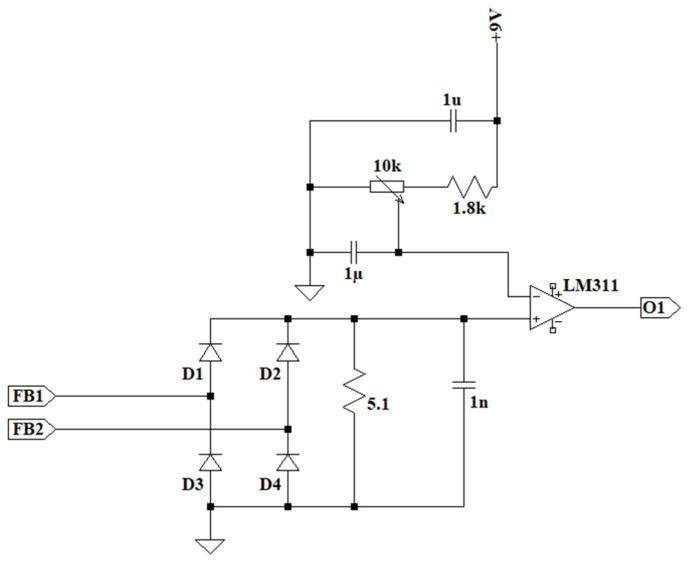
Primary overcurrent sensor with regulated current reference from a potentiometer.

In the case of the overcurrent detector, the 1 A signal is rectified using a full bridge, and this direct current signal passes through a 5.1 Ω resistor, creating a voltage of 5 V. By employing a voltage comparator with a variable reference signal of up to 9 V, the system’s overcurrent can be regulated in a range of 0–1800 A. The simplified electrical schematic diagram can be seen in Figure 11.

### 2.5. System Control

The control of the prototype is implemented through a ZCS system that adjusts the resonance frequency to the natural resonance frequency of the primary circuit, in this case of 37 kHz [33]. This allows for switching the IGBT transistors at the zero crossing of the current, reducing switching losses to a value close to zero [34]. This control stage is implemented using feedback from the primary circuit’s current input. Feedback transformers supply the current sensor directly generating a trigger signal as shown in Figure 10 when the current crosses zero. This is achieved by employing a high-speed comparator that acts as the reference signal, which is then sent to the IGBT drivers as an on/off control signal. This control is performed in open-loop mode, as the use of high-speed integrated circuits minimizes the delay between the current zero-crossing and the triggering of the IGBTs. This delay only impacts system stability at significantly higher frequencies, where the signal delay becomes critical.

**Figure 12:**
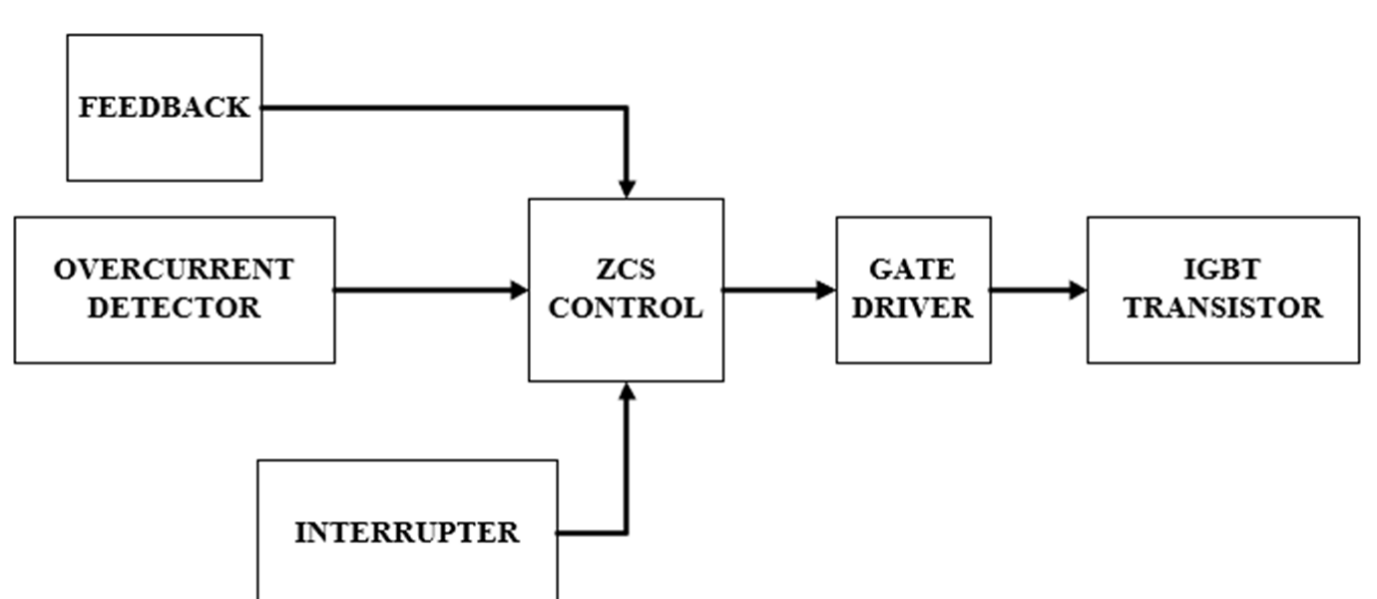
Control circuit diagram for Tesla Coil.

The control stage is also responsible for taking the overcurrent signal from the sensor and, when triggered, ceases to switch at the next current zero crossing. Therefore, in the event of a critical failure, the system must withstand the fault current for at least one cycle without damaging the system. The duty cycle of the Tesla Coil is not 100%. Therefore, it requires an external switching device to control the on-time. This device is called a switch and is simply a microcontroller with a potentiometer that sends the signal via fiber optics. The control stage generates two identical but opposite 5 V signals to the drivers to control the transistors. An example of the complete system operation can be seen in Figure 12.

### 2.6. Drivers

When switching currents and frequencies that significantly exceed the manufacturer’s recommendations, the gates of the IGBT transistors require switching assistance. Therefore, the prototype employs a gate supply of ±24 V, allowing for faster switching by charging and discharging the gate more efficiently [35]. Under normal conditions, drivers for these IGBTs provide a voltage of ±20 V, and it is quite common to use only a +20 V signal for activation, without a bipolar configuration, using a value of 0 V for the off state.

In this case, the supply is bipolar with a higher voltage. This is achieved using a specific driver for IGBTs, powered by a control circuit that generates a square wave signal based on feedback from the current sensor. The signal from the control circuit feeds small drivers connected to the gates of several FDD8424 type MOSFETs, which are used as amplifiers to generate the ±24 V signal and a maximum current of up to 40 A to power the gates of the IGBT transistors.

**Figure 13:**
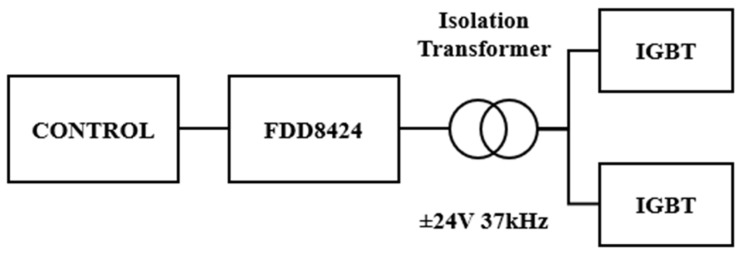
Gate driver circuit using FDD8424 MOSFETs and isolation transformer for two IGBT.

Since it is a resonant circuit with an H-bridge power supply, the transistors operate in pairs of two. To take advantage of the driver’s high current capability, a high-frequency magnetic core transformer is used to supply the gates of the MOSFET pair with a bipolar signal of ±24 V. This transformer has a transformation ratio of 1:1, and with a 50% duty cycle in operation, it avoids any unusual behavior. To avoid damage to the transistor gate, a voltage limiter circuit of 33 V is used along with a gate resistor of 4.7 Ω, as shown in Figure 13. This way, switching is performed much faster by charging and discharging the gate with higher potential difference. Therefore, it is possible to increase the system frequency but requiring more current at the transistor gate [18].

**Figure 14:**
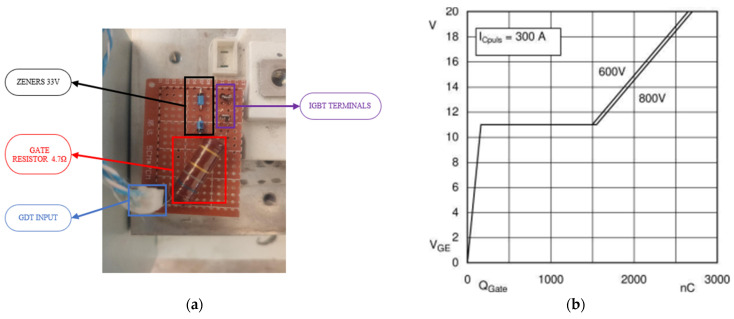
IGBT Transistor Driver. (**a**) Electrical circuit of the gate input from the driver with protection diodes and gate resistor. (**b**) Gate charge of an SKM transistor in nC with gate voltage and 300 A collector current.

The circuit assembly has been completed on a board with all the auxiliary components together, as shown Figure 14a. The nominal current of the driver required for the switching of each transistor can be calculated in a simplified manner (1) using the gate capacitance provided by the manufacturer in Figure 14b for the SKM under normal operating conditions and the switching times.

(1) $$I_{G}=\frac{Q_{g}}{t_{sw}}=\frac{2700\;nC}{27\;µs}=100\;mA$$

In the case of the maximum peak current of the driver, it is simplified by assuming that the internal resistance is close to zero. Therefore, at the initial moment, to switch the transistor from +24 V to −24 V, a current of 10.21 A will be required, as shown in (2). In this case, the driver is capable of supplying a transient current of up to 40 A.

(2) $$I_{Gpico}=\frac{U_{Gon}-U_{Goff}}{R_{Ginter}+R_{Gextern}}=\frac{24-\left(-24\right)V}{0\;\Omega +4.7\;\Omega }=10.21\;A$$

**Figure 15:**
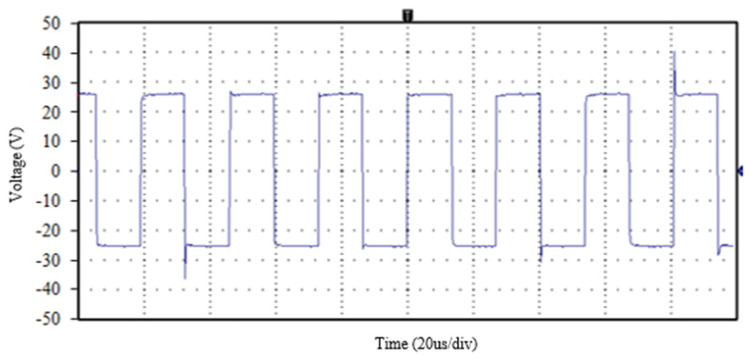
Bipolar IGBT trigger signal measured with an oscilloscope at the output of one GDT.

Using an oscilloscope connected to the output of the driver, the effect of the bipolar gate supply signal can be observed, as shown in Figure 15.

## 3. Model and Simulation

The objective of the modeling is to obtain the transfer function between the output voltage of the inverter and the current in the primary circuit that flows through the transistors [32].

### 3.1. Resonant Circuit Model

**Figure 16:**
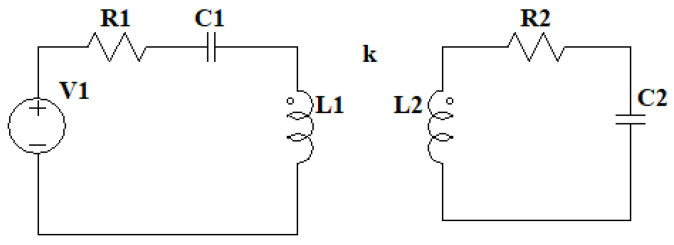
Double resonant circuit equivalent model 1, primary circuit at the left and secondary circuit at the right coupled by a k factor.

The behavior of the two resonant circuits is based on energy transfer through magnetic coupling (Figure 16).

**Figure 17:**
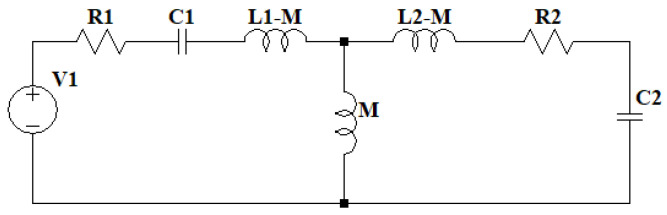
Double resonant circuit equivalent model 2, with mutual inductance of the primary and secondary circuit.

**Figure 18:**
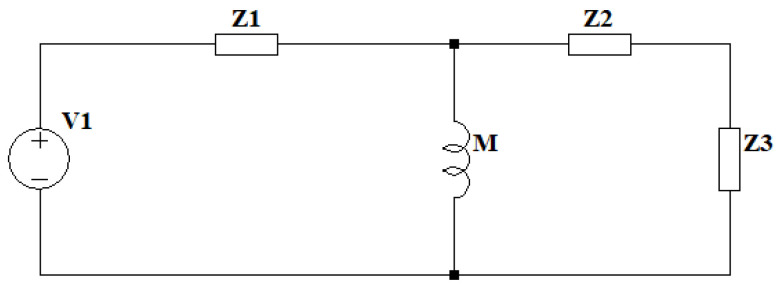
Double resonant circuit equivalent model 3 with simplified impedances and output impedance as Z3.

The model of the two resonant circuits is similar to that of a real transformer, with the distinction that it lacks a magnetic core; instead, as it is a Tesla coil, the core is air. This allows for the use of the same simplifications as those seen in [36,37], assuming that both iron losses and magnetizing reactance are negligible. To couple the two resonant circuits, the parameter M, corresponding to mutual inductance, is introduced, representing the influence of the primary circuit on the secondary and vice versa, as shown in Figure 17. By combining the resistances, inductances, and capacitances of the elements into two impedances Z1 and Z2, the circuit can be simplified, except for impedance Z3, corresponding to the capacitor, where the transformer’s output voltage can be considered, as illustrated in Figure 18.

The values of the simplified impedances are given by Equations (3)–(6).

(3) $$Z_{1}=R_{1}+\frac{1}{C_{1}s}+L_{1}s-M$$

(4) $$Z_{2}=R_{2}+L_{2}s-M$$

(5) $$Z_{3}=\frac{1}{C_{2}s}$$

(6) $$M=k\sqrt{L_{1}L_{2}}\cdot s$$

### 3.2. Output Current

**Figure 19:**
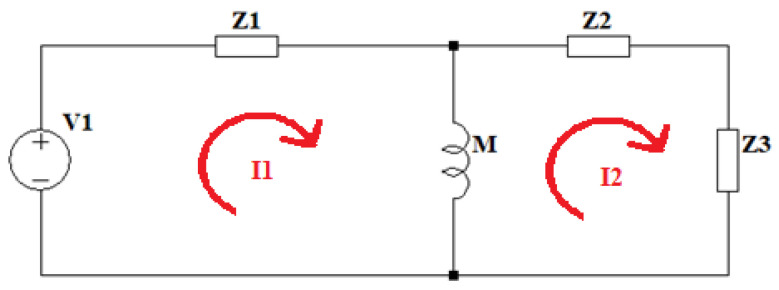
Double resonant circuit equivalent model 4 current analysis.

The presented prototype is based on the overcurrent of the IGBT transistors, so the required transfer function for the analysis is the one that relates the input voltage to the current in the primary resonant circuit. The development of the currents in the two loops of the simplified circuit (Figure 19) allows for obtaining the required Equation (7).

(7) $$\frac{I_{1}}{U_{1}}=\frac{1}{{\frac{M^{2}}{M+Z_{2}+Z_{3}}-Z}_{1}-M}$$

### 3.3. Model Losses

**Figure 20:**
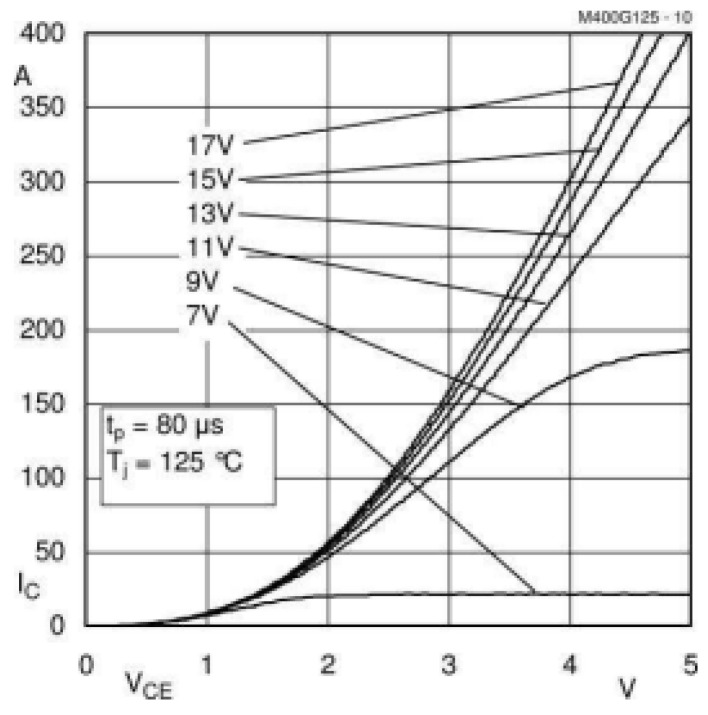
Collector–emitter voltage of SKM400 with gate voltage and collector current.

Most of the losses in IGBTs are due to conduction and switching losses of the transistors. The conduction losses in an IGBT transistor are determined by the RMS current flowing through the transistor and the voltage drop across its terminals. Since the losses depend on the characteristics of the IGBT, the SKM400 characteristics provided by the manufacturer have been used. In Figure 20, the collector–emitter voltage of the transistor is shown; since very high currents are being used, a voltage of 5 V will be assumed. Additionally, an internal resistance of 6.3 mΩ, as specified by the manufacturer, has been considered.

With these values and assuming a peak current of 1000 A, a conduction time of 10%, and considering that the inverter topology is a full bridge, the RMS current that the transistor will need to withstand can be estimated, as shown in Equation (8).

(8) $$I_{RMS}=D\cdot \frac{I_{peak}}{\sqrt{2}}=0.5\cdot 0.1\cdot \frac{1000}{\sqrt{2}}=70.71\;A$$

Thus, the conduction losses in the system can be calculated using the collector–emitter voltage and internal resistance, as shown in Equation (9).

(9) $$P_{d}=0.5\cdot \left(I_{RMS}\cdot U_{CE}+{I_{RMS}}^{2}\cdot R_{C}\right)\;=0.5\cdot \left(70.71\;A\cdot 7\;V+{\left(70.71\;A\right)}^{2}\cdot 6.3\;m\Omega \right)\;=262.98\;W$$

On the other hand, the switching losses are given by Equations (10)–(12). These losses primarily depend on the switching frequency.

(10) $$E_{on}=\int_{t1}^{t1+tsw(on)}U_{CE}(t)\cdot I_{C}(t)dt$$

(11) $$E_{off}=\int_{t2}^{t2+tsw(off)}U_{CE}(t)\cdot I_{C}(t)dt$$

(12) $$P_{sw}=D\cdot f_{sw}\cdot (E_{on}+E_{off})$$

In this work, calculating the switching losses mathematically is a complicated task, therefore, a simulation model based on Simulink R2024b is employed. This model is also used to estimate conduction losses with greater accuracy, reflecting more realistic conditions. Additionally, the junction temperature of the IGBT transistors is calculated using thermal analysis based on the Foster thermal model [38].

**Figure 21:**
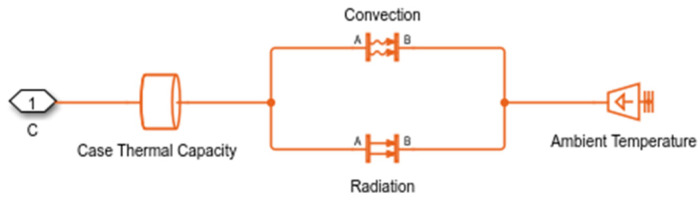
Simulink thermal model based on case thermal capability and heatsink with constant ambient temperature.

**Figure 22:**
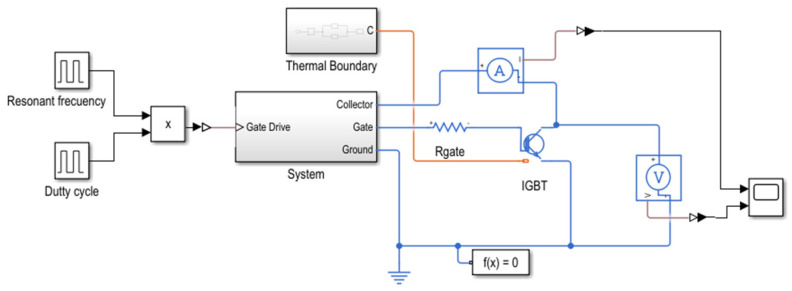
Losses and junction temperature simulation in Simulink model based on datasheet carasteristics.

To develop the transistor model, an RLC load resonating at the same frequency as the Tesla coil (37 kHz) is integrated into the system. The transistors are modeled using parameters specified in the manufacturer’s datasheet, including switching losses, turn-on and turn-off losses, and collector–emitter voltages. For the thermal model simulation, Simulink is also utilized, coupling the heatsink model to the transistor with specifications such as the number and dimensions of fins and installation characteristics, as shown in Figure 21. The complete model can be seen in Figure 22.

**Figure 23:**
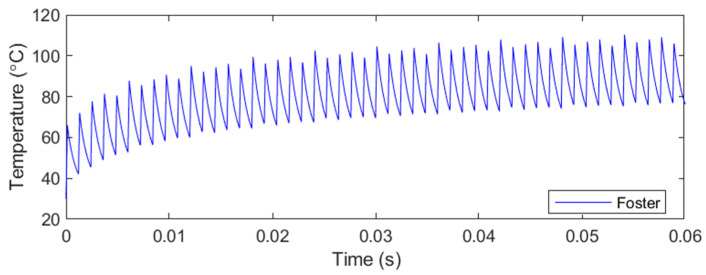
Simulink simulation of junction temperature of the IGBT based on Foster model at a 37 kHz resonant frequency and 10% duty cycle.

The thermal and loss model is simulated using various simulation times and a duty cycle of 10%, as would be expected under real operating conditions. The results obtained correspond to a single transistor with a peak current of 1000 A at 37 kHz and an operation time sufficient for the junction temperature to stabilize. Figure 23 presents the results of the transistor’s thermal simulation, showing that a junction temperature of 96 °C is reached in a 10-min simulation for the IGBT.

**Figure 24:**
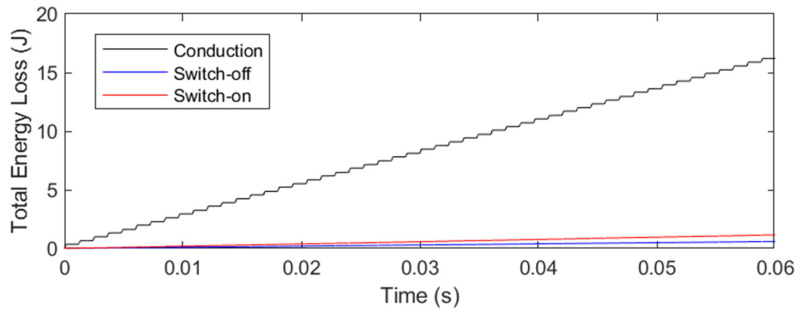
Simulink simulation of switching on and off losses based on the characteristic of the IGBT transistor at a 37 kHz resonant frequency and 10% duty cycle.

Additionally, Figure 24 displays the transistor losses, both conduction and switching (turn-on and turn-off) losses. In a 10-min simulation, the transistor exhibited conduction losses of 276.11 W and switching losses of 29.19 W. It is noteworthy that the conduction losses in the model slightly exceed the ideal losses calculated using Equation (9). Moreover, due to the ZCS system, the switching losses are significantly reduced compared to the conduction losses and, in some cases, could be considered negligible. If this system were implemented without ZCS, switching losses would be considerably higher.

### 3.4. Spice Simulation

**Figure 25:**
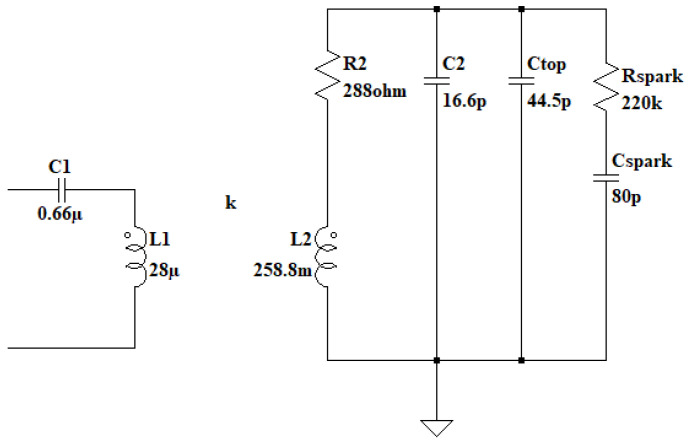
Simulation model of resonant Tesla coil based on the primary and secondary inductance, capacitance of the coil and topload and a RC model of the spark.

The complete model of the dual resonant system can be seen in Figure 25. In this model, the secondary circuit and the electric arc have been represented as a series of capacitors to resemble the real circuit.

All parameters of the system have been measured using various methods and instruments. These parameters are presented in Table 1.

**Figure 26:**
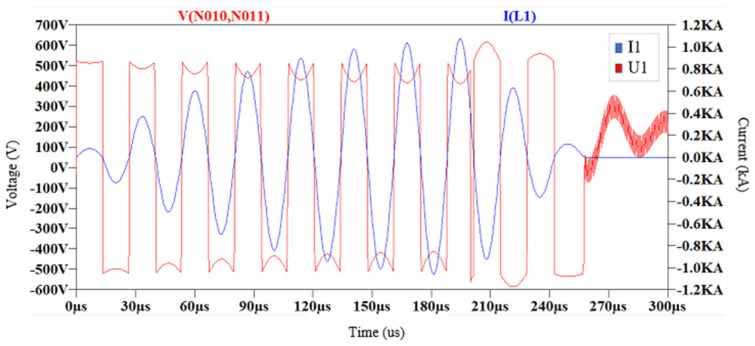
Simulation of the circuit, voltage applied in red and primary circuit current in blue with ZCS control.

**Figure 27:**
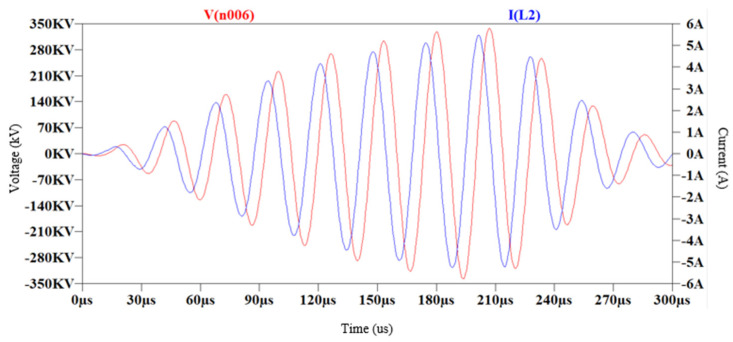
Simulation of the circuit, with voltage generated in the secondary circuit in red and current in the secondary circuit in blue.

The transfer function allows for modeling the current in the resonant circuit based on a square voltage input with a 50% duty cycle and conduction times of 250 µs at a frequency of 37 kHz, simulating the effects of IGBT transistor switching. The simulation results on LTSpice XVII for the primary resonant circuit conclude that the currents in ZCS are very high, on the order of 1000 A for the given circuit parameters, as shown in Figure 26 in blue. Furthermore, the transfer of currents and voltages in the secondary circuit can also be observed. In Figure 27, the voltage in red and the current in blue are generated gradually due to the low coupling coefficient.

## 4. Results

The operation of the prototype allows for determining the maximum currents flowing through the IGBT transistors under ZCS conditions in the primary resonant circuit.

### 4.1. Prototype Test

**Figure 28:**
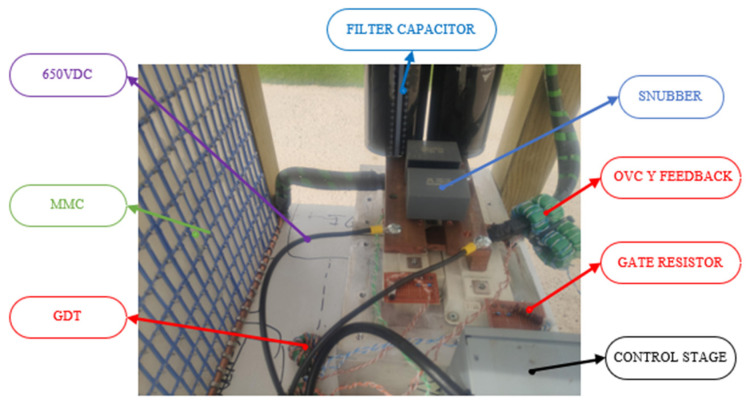
Schematic of the primary resonant circuit and control based on low inductance design with a MMC capacitor.

For the measurements on the Tesla coil, a digital oscilloscope Instrustar ISDS205C is used with a range of 1 V/div and 0.1 V/div and a measurement error for these ranges of 5%, the range of time measurement is 20 µs/div and 100 µs/div with a measurement error of 5%. The prototype has been powered with a voltage of 521 V and a frequency of 37 kHz. Measurements are taken using two current transformers in cascade with a transformation ratio of 1:1024 connected to a 5 Ω resistor, thereby measuring the voltage drop across it with the oscilloscope. Also, the voltage across the IGBT collector–emitter has been measured. The components of the circuit can be seen in Figure 28.

**Figure 29:**
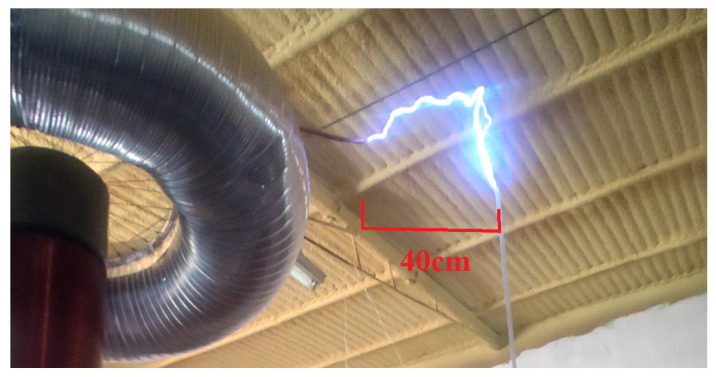
High voltage discharges of 40 cm at the secondary from the topload to ground tube.

As this is a solid-state Tesla coil operating at a resonance frequency adjusted between the primary and secondary circuits, the energy generated in the primary resonant circuit is transferred to the secondary resonant circuit through magnetic coupling and is ultimately discharged in the form of an electric arc. Since the voltage levels produced are very high, the electrical discharges extend several centimeters in length, exceeding tens of kV, as shown in Figure 29. Because these voltages and currents could not be directly measured due to their high magnitude, they are estimated using simulation data. These voltages are in the range of 200 kV according to the model of the electric arc used, while the currents in the secondary circuit are on the order of milliamperes.

In this case, the input voltage is reduced, resulting in a current in the primary resonant circuit on the order of hundreds of amperes. By utilizing the variable power supply, electric arcs of greater length and current can be achieved, reaching up to 1000 A transiently with transistors rated for a nominal current of 300 A at 80 °C.

### 4.2. Operating Currents

**Figure 30:**
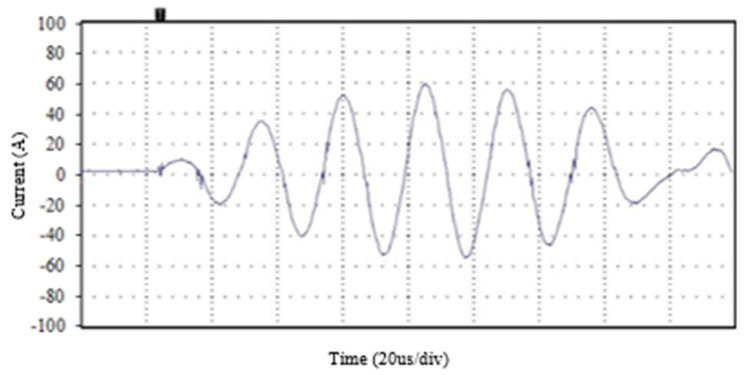
Initial test of 60 A low current at the primary circuit and 120 µs conduction time measured with an oscilloscope and transformer.

The operation of the system is conducted in a controlled manner, progressively increasing the currents to analyze the maximum current capacity of the IGBT transistors under ZCS conditions. Initially, the conduction times were set to 120 µs, and the voltage is gradually increased, resulting in currents of 60 A, confirming that the system worked with no damage, as shown in Figure 30.

**Figure 31:**
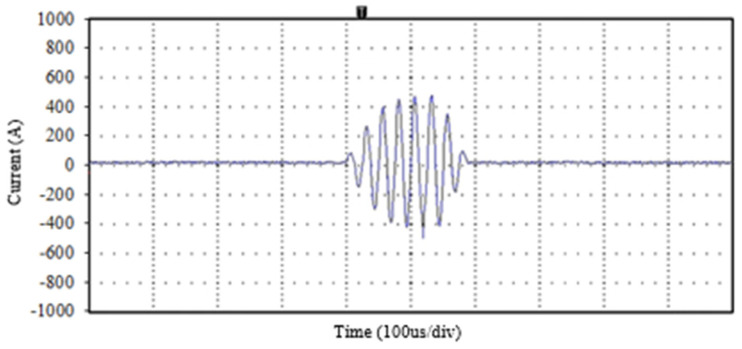
Test with 400 A current at the primary circuit exceeding the nominal steady-state conditions of the transistors.

Subsequently, the currents were increased to 400 A, exceeding the nominal ratings of the transistor under steady-state conditions without causing any damage. These currents can be seen Figure 31

**Figure 32:**
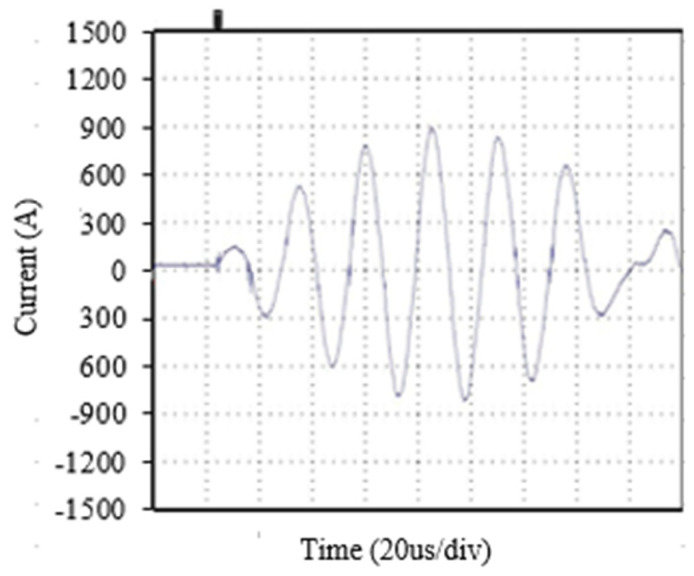
Maximum transient currents of 900 A in the IGBT transistors with a supply voltage of 650 V at the primary capacitor.

The limits of the maximum transient current for the transistor model are specified as 600 A by the manufacturer. This limit is exceeded by more than 150% when powering the system with voltages of 650 V, resulting in currents of 900 A without any damage occurring at any point, as shown Figure 32.

**Figure 33:**
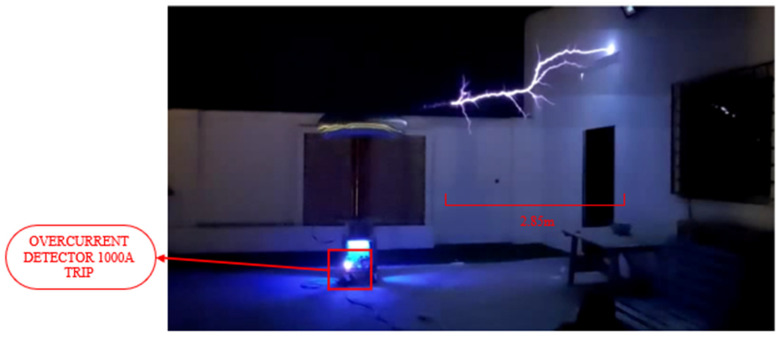
Operation of the Tesla coil with the overcurrent detector triggered at 1000 A and 2.85 m of spark from topload to ground.

For the safety of the measurement elements, the currents did not exceed 900 A. However, by utilizing the overcurrent detector along with the measurement sensor coupled to the resonant circuit, the current is increased to achieve 1000 A without damaging the transistors, sustaining several minutes of operation, as shown in Figure 33. The IGBT conditions were 80 °C and 37 kHz frequency.

### 4.3. Final Analysis

**Figure 34:**
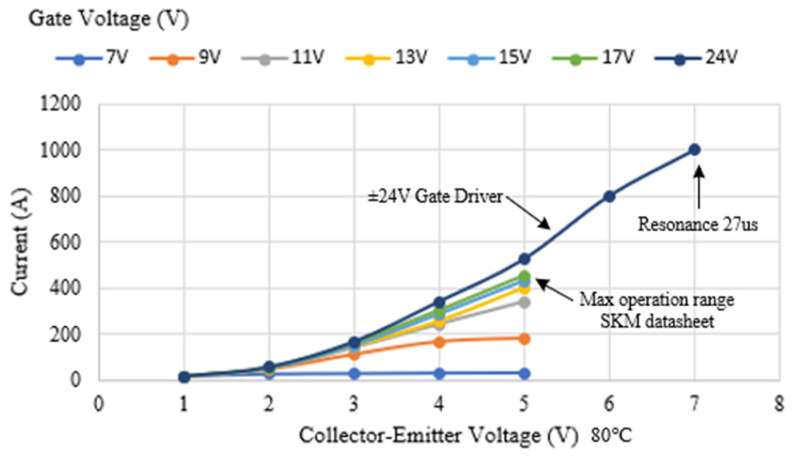
Overcurrent to normal operation comparation of the current and collector–emitter voltage of SKM400 transistors.

For the analysis of the operating currents in this work, the currents of the SKM400 under resonance conditions are compared with the nominal conditions of the transistor at different gate supply voltages, in our case, ±24 V. These results are shown in Figure 34, where it can be observed that when the gate voltage increases and becomes bipolar, the currents may transiently exceed the nominal values, despite having a higher collector–emitter voltage.

**Figure 35:**
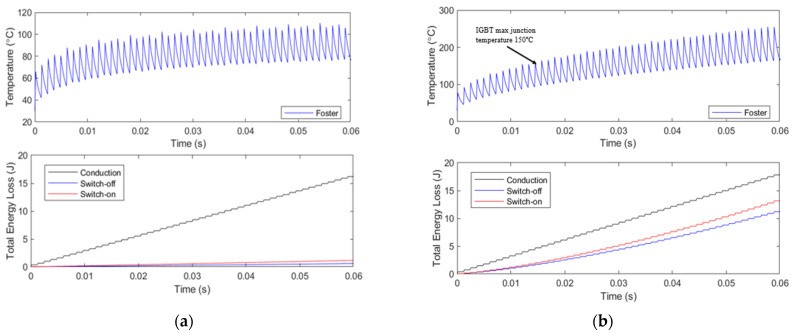
Junction temperature and losses comparation. (**a**) Simulink model with ZCS control system of the IGBT at 37 kHz. (**b**) Simulink model of a IGBT at 37 kHz without ZCS system, junction temperature absolute maximum reach at 0.015 s simulation time.

The thermal and loss simulations of the Tesla coil system, as described in Section 2.2, are compared with an identical model that does not employ a ZCS system. In this alternative model, the turn-on and turn-off of the IGBT transistor do not occur precisely at the zero-crossing point, resulting in a significant increase in losses. As shown in Figure 35a, the temperature remains within the safe margin, while in Figure 35b, without ZCS, the switching losses increase significantly, leading to a junction temperature exceeding 150 °C within 0.015 s, which would destroy the transistor.

Furthermore, these results have been compared with those obtained in similar studies, such as [25], where short-circuit currents exceed nominal levels during transistor turn-off in the event of faults in a SiC IGBT transistor model. In this work, similar overcurrents are achieved, reaching 170% of the device’s peak current for both the transistor’s turn-on and turn-off phases. In the case of [25], the overcurrents reached 184% during turn-off, but this led to the complete destruction of the IGBT transistor through theoretical simulation. Additionally, in this work, a controlled overcurrent is successfully maintained for several pulses over a period of 10 min without causing degradation of the transistor or loss of functionality. The conduction times of the transistors are also similar; in this work, the transistor’s turn-on time is 13.5 µs, sustained over multiple pulses, whereas in [25], conduction time before simulated destruction occurs at 15 µs.

Therefore, the 1000-A current achieved during the prototype tests demonstrates the feasibility of overdriving IGBT transistors by more than 300% of their nominal current and 170% above the peak currents specified by the manufacturer. Also the overcurrent capability of IGBT transistors is verified not only during turn-off, as in [25], also during turn-on in a repetitive mode without causing damage to the IGBT transistor without causing destruction.

This clearly shows that the currents in IGBT transistors can be significantly transiently exceeded by employing ZCS techniques along with appropriate gate control circuits that enhance the switching capabilities of these transistors, reducing switching losses to values very close to zero. It is noteworthy that the peak power achieved by the transistors is around 650 kW.

## 5. Conclusions

In this work, a doubly resonant circuit has been implemented to demonstrate the feasibility of using IGBT power transistors at currents significantly higher than those permitted by manufacturers, thereby reducing component selection costs. This has been achieved through the use of soft-switching techniques such as ZCS, which, as seen in other studies, minimize switching losses, allowing the transistor current to approach the maximum recommended levels. In this case, it has been possible to triple the maximum current of the transistor specified by the manufacturer.

## Figures and Tables

**Table 1:** Model parameters.

Parameter	Value
$C_{TOP}$	44.5 pF
$C_{2}$	16.6 pF
$C_{1}$	0.66 uF
$C_{SPARK}$	80 pF
$R_{SPARK}$	220 kΩ
$R_{1}$	0.1 Ω
$R_{2}$	288 Ω
$L_{1}$	28 uH
$L_{2}$	258.8 mH
k	0.144
$U_{1}$	521 V

## Data Availability

The data presented in this study are available on request from the corresponding author. The raw/processed data needed to reproduce these findings cannot be shared publicly at this time, as they are also part of an ongoing study.
